# Nanocarriers of Fe_3_O_4_ as a Novel Method for Delivery of the Antineoplastic Agent Doxorubicin Into HeLa Cells *in vitro*

**DOI:** 10.3389/fonc.2019.00250

**Published:** 2019-04-10

**Authors:** Kun-kun Xia, Yong Lyu, Wei-tang Yuan, Gui-xian Wang, Harrison Stratton, Shui-jun Zhang, Jie Wu

**Affiliations:** ^1^Department of Hepatobiliary and Pancreatic Surgery, First Affiliated Hospital of Zhengzhou University, Zhengzhou, China; ^2^Department of Colon and Rectal Surgery, First Affiliated Hospital of Zhengzhou University, Zhengzhou, China; ^3^Department of Neurobiology, Barrow Neurological Institute, St. Joseph's Hospital and Medical Center, Phoenix, AZ, United States

**Keywords:** redox-responsive, Fe_3_O_4_, nanocarriers, drug delivery, HeLa cells

## Abstract

Here we report the synthesis and *in vitro* characterization of a redox-sensitive, magnetically inducible nanoparticle carrier system based on the doxorubicin (DOX) drug delivery model. Each quantal nanocarrier unit consists of a magnetite Fe_3_O_4_ nanoparticle core that is further encapsulated in self-assembled micelles of the redox-responsive polyethylene glycol derivative, DSPE-SS-mPEG. The nanocarrier system was prepared using a combination of ultrasonication and dialysis to produce the microenvironment sensitive delivery system. The final synthesized and DOX-loaded magnetic nanocarriers had an average size of ~150 nm when assembled with a 6.9% DOX payload. The release rate of DOX from these redox-responsive magnetic nanocarriers was shown to be accelerated *in vitro* when in the presence of glutathione (GSH). Furthermore, we demonstrated that more redox-responsive magnetic nanocarriers could be taken up by HeLa cells when a local magnetic field was applied. Once internalized within a cell, the micelles of the outer nanocarrier complex were broken down in the presence of higher concentrations of GSH, which accelerated the release of DOX. This produces a particle with dual operating characteristics that can be controlled via a specific cellular environment coupled with an exogenously applied signal in the form of a magnetic field triggering release.

## Introduction

Chemotherapy is the most commonly used approach to treating cancer. Traditionally, the chemotherapeutic agents (doxorubicin, paclitaxel, etc.) are systemically delivered through intravenous injection. While this is often an effective approach and can successfully eliminate malignant cell populations, treatment-associated morbidity is often significant (1). Quite frequently this is a result of unintended action of the therapeutic agent at non-specific cellular targets causing injury to healthy somatic cells in addition to the desired effect on malignant cells (2–6). Despite this large unintended effect on healthy cells of the patient, chemotherapy remains a pillar of cancer treatment due to its efficacy, particularly when used as part of a multimodal treatment plan. At the intersection of the potency of chemotherapy as a curative agent and the extensive side effect profile causing wide-ranging cytotoxicity lays a rationale that suggests transport of the chemotherapeutic agent directly to the tumor site, which avoids systematic exposure, may alleviate unintentional cytotoxic effects on healthy tissue. This concept has existed in the medical literature for quite some time, but only recently has progress in functionalization of mesoscopic carrier particles led to significant progress in realizing this goal. There are now several readily available preparations for a medical oncology approach to cancer treatment that utilizes nanotechnology, in the form of nanoparticle assemblies, to facilitate the transport of highly potent cytotoxic compounds more selectively into tumor sites with restricted systemic circulating concentrations (7–9). These nanoparticles can be constructed such that they resist degradation or internalization except at the target tissue of interest where they are then able to deposit and release their payload at the site of the malignancy and not in healthy tissue (10–12). These particles can also be used to focus energy from external radiative sources into tumor masses acting to physically damage the cancerous cells in addition to the chemical damage affected by the pharmacological agent (13). This is an elegant solution to the problem of how to transport chemotherapeutic drugs to the tumor site without leakage and subsequently release a drug into the tumor-specific microenvironment is an important issue that needs to be solved in the treatment of cancer. The rise of nanotechnology has provided a new set of tools for use in solving this problem of targeted drug delivery (14–17).

The use of nanoparticles as a carrier vehicle for the targeted delivery of chemotherapeutic drugs has the potential to greatly reduce collateral damage to non-cancerous human tissues and organs (18, 19). For example, by modifying the surface of a nanoparticle with intelligent molecules, the nano drug carriers can stimulate drug release in response to the particular micro-environment of pathological tissues to reduce the incidence of healthy cell damage and selectively kill cancer cells (20–22). The study of nano drug carriers provides a new direction for the delivery of care in addition to the traditional cancer treatment approaches already in use and possesses significant potential for future clinical applications (20, 23, 24). In this study, we constructed spherical nanoparticle carriers containing doxorubicin (an antineoplastic drug) with a diameter of about 150 nm. We provide functional data to demonstrate that the entry of the drug carriers into HeLa cells can be enhanced in a magnetic field and the release of the drug can be facilitated by elevating the concentration of glutathione (GSH), resulting in the demise of HeLa cells. As several cancer cells have high intracellular GSH concentrations, using the constructed nanoparticle carriers may achieve satisfying efficacy in killing cancer cells, while causing only minor damage in normal tissue (25–27).

## Materials and Methods

### Materials

The Fe_3_O_4_ nanoparticles were prepared using a thermal decomposition method described previously (28). DSPE-SS-mPEG 2000 was purchased from Xi'an Ruixi Biotechnology Co. (Xi'an, China), Doxorubicin hydrochloride (DOX·HCl) and GSH were purchased from Sigma-Aldrich (St. Louis, MO, USA), dimethyl sulfoxide (DMSO) and triethylamine (TEA) were obtained from Shanghai Chemical Co. (Shanghai, China) (29).

Human cervical adenocarcinoma (HeLa) cells were purchased from the China Center for Type Culture Collection (Wuhan University) and cultured in Dulbecco's modified Eagle's medium (DMEM, Gibco Life, Grand Island, NY, USA) supplemented with 10% fetal bovine serum (FBS, HyClone, Logan, UT), 2 × 10^−3^ M L-glutamine and 1% antibiotics mixture (10,000 U of penicillin and 10 mg of streptomycin) (Gibco). The cells were incubated in a humidified atmosphere containing 5% CO_2_ at 37°C.

### Preparation of Nanocarriers

DOX-loaded redox-responsive magnetic nanocarriers were prepared using an ultrasonication-dialysis method. Briefly, DOX·HCl (10 mg) was stirred in DMSO (5 mL) with twice the number of mole of TEA for 2 h to obtain the DOX base. 80 mg of DSPE-SS-mPEG was added to the solution, which was stirred at room temperature for another 2 h. Meanwhile, the Fe_3_O_4_ nanoparticles (20 mg) were dissolved in 10 mL of (tetrahydrofuran) THF. The above two solutions were mixed and added to ultrapure water (25 mL) with ultrasonication. The mixed solution was then transferred into a dialysis tube and dialyzed against ultrapure water for 48 h at room temperature. Similarly, DOX-free nanocarriers were prepared using the above mentioned protocol without the addition of DOX.

### Characterization of Nanocarriers

The size of the nanocarriers in aqueous solution was measured using a Zetasizer analyzer (Malvern Zetasizer Nano, Zen 3690+MPT2, Malvern, UK). Ultrastructural features and surface geometry of the synthesized nanocarriers was observed by transmission electron microscopy (TEM) (Tecnai G2 F20 S-TWIN electron microscope, FEI company. the USA) at an accelerating voltage of 200 kV.

DOX-loaded nanocarriers were dissolved in DMSO to determine the total content of loaded drug. The DOX content in DMSO was determined by high-performance liquid chromatography (HPLC, Agilent) using a calibration curve obtained from DOX/DMSO solutions containing a known concentration of DOX.

For Fe_3_O_4_ content measurement, the weighed, freeze-dried nanocarriers were digested in a 1 M HCl solution. The resulting digestion product was then analyzed for atomic species using inductively coupled plasma-atomic emission spectroscopy (TCP-AES, Thermo Electron, USA).

### Redox-Triggered Disassembly of Nanocarriers

The change in the size of redox-responsive magnetic nanocarriers in response to 20 mM GSH in PBS (0.01 M, pH 7.4) was measured using dynamic light scattering (DLS). Briefly, 20 mM GSH was added to 1.5 mL of PBS containing nanocarriers within a glass cell. The solution was then placed in a shaking water bath at 37°C, oscillating at 150 rpm. At varying intervals following agitation, the size of nanocarrier particles contained in solution was assessed using DLS.

### *In vitro* Redox-Triggered Release of DOX From DOX-Loaded Nanocarriers

The *in vitro* release profile of nanocarriers was investigated using dialysis of DOX-loaded nanocarriers in two different media: PBS or PBS supplemented with 20 mM GSH. Each solution was diluted to 1.5 mg/mL and 5 mL of the solution was transferred into a membrane tubing. The tubing with the solution was immersed in a tube containing 50 mL of the buffer solution in a shaking water bath at 37°C to acquire the “sink” condition. At predetermined intervals, 20 mL of the external buffer was withdrawn and replaced with a fresh solution of the corresponding buffer. The amount of DOX released was determined using HPLC.

### Cell TEM Imaging

For TEM imaging, HeLa cells were incubated with DOX-loaded nanocarriers at a final DOX concentration of 5 μg/mL in DMEM for 2 h at 37°C in the presence or absence of an externally applied magnetic field. The culture medium was removed and the cells were pre-fixed with 2.5% glutaraldehyde in PBS at 4°C for 2 h and post-fixed with 1% osmium tetroxide in PBS at 4°C for 2 h. The cells were then dehydrated using serially increasing concentrations of ethanol and flat embedded in Epon 812. After polymerization at 60°C for 48 h, ultrathin sections (60–80 nm) were trimmed and further stained with uranyl acetate and lead citrate. Micrographs of the stained samples were collected with an FEI Tecnaio G220 TWIN Transmission Electron Microscope.

### Cell Viability Assay

To evaluate the anti-tumor activity of DOX-loaded nanocarriers, the cytotoxicity of DOX-loaded nanocarriers or free DOX against HeLa cells was evaluated *in vitro* using the MTT assay. HeLa cells were seeded into a 96-well plate at a density of 4.0 × 10^3^ cells/well in 100 μL of complete DMEM. The cells were cultured for 24 h at 37°C in a 5% CO_2_ atmosphere. Subsequently, the cells were incubated with DOX-loaded nanocarriers or free DOX for 24 h at 37°C with or without the presence of an external magnetic field. DOX-loaded nanocarriers or free DOX were diluted in complete DMEM to a final DOX concentration ranging from 0.4 to 40 μg/mL. After the incubation, 10 μL of MTT solution (5 mg/mL in PBS 7.4) was added to each well and incubated for 4 h. The media with MTT solution was removed and 200 μL of DMSO was added to dissolve the formazan crystals and further incubated for 15 min at 37°C. The absorbance readings were recorded using a microplate spectrophotometer (PowerWave XS2, BioTek Instruments, USA) at a wavelength of 540 nm. The cell viability was normalized to that of cells cultured in complete DMEM. The dose-effect curves were plotted and data are presented as the average ± SD (*n* = 4).

### Confocal Laser Scanning Microscopy (CLSM) Observation

CLSM was used to examine the intracellular distribution of DOX. HeLa cells were seeded on coverslips in the wells of a 24-well plate at a density of 4.0 × 10^4^ cells/well in 1 mL of complete DMEM. The cells were incubated for 24 h at 37°C in a 5% CO_2_ atmosphere. The cells were incubated with DOX-loaded nanocarriers at a final DOX concentration of 5 μg/mL in DMEM for 2 h at 37°C with or without an external magnetic field. After removal of the medium, the cells were washed three times with cold PBS, fixed with 1 mL of 4% paraformaldehyde for 30 min at 4°C, and stained with 2-(4-amidinophenyl)-6-indolecarbamidine dihydrochloride (DAPI, Roche) for 10 min. Finally, the slides were mounted with 10% glycerol solution and viewed using a LeicaTCS SP8 (Leica Microscopy Systems Ltd., Germany).

## Results and Discussion

### Characterization of Nanocarriers

**Figure 1:**
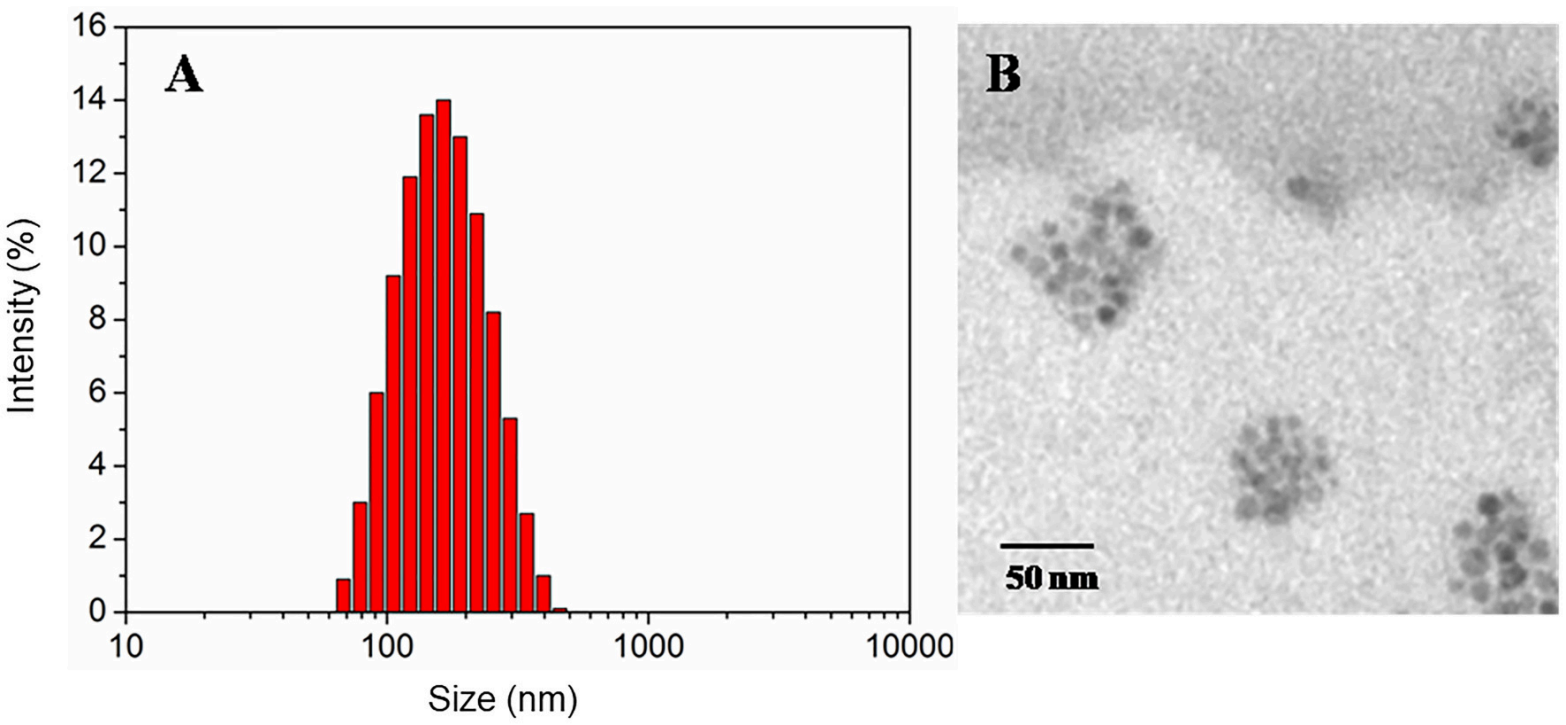
The size distribution by DLS **(A)**, and TEM micrographs **(B)** of redox-responsive magnetic nanocarriers.

The particle size and polydispersity (PDI) of DOX-free or DOX-loaded nanocarriers were determined by DLS, as shown in Table 1. The prepared DOX-free nanocarriers and DOX-loaded nanocarriers (Figure 1A) were determined to be 131 or 150 nm respectively, with a narrow size distribution, thereby making them suitable as anticancer drug carriers. The morphology of the redox-responsive magnetic nanocarriers was observed using TEM. Figure 1B shows the morphology of the nanocarriers. Because DSPE-SS-mPEG does not significantly attenuate electron scattering under TEM, nanocarriers are largely present as isolated clusters of Fe_3_O_4_ nanoparticles with a spherical shape. The drug loading content values of DOX-loaded nanocarriers was 4.6% (Table 1). Whereas, the Fe content of DOX-free or DOX-loaded nanocarriers was 14.7 and 13.3%, respectively (Table 1).

**Table 1:** Properties of DOX-free and DOX-loaded nanocarriers.

**DOX-free nanocarriers**			**DOX-loaded nanocarriers**			
**Size (nm)**	**PDI**	**Fe content (wt%)**	**Size (nm)**	**PDI**	**Fe content (wt%)**	**PLC (wt%)**
131	0.26	14.7	150	0.19	13.3	4.6

### The Redox-Responsive Stability of Nanocarriers

**Figure 2:**
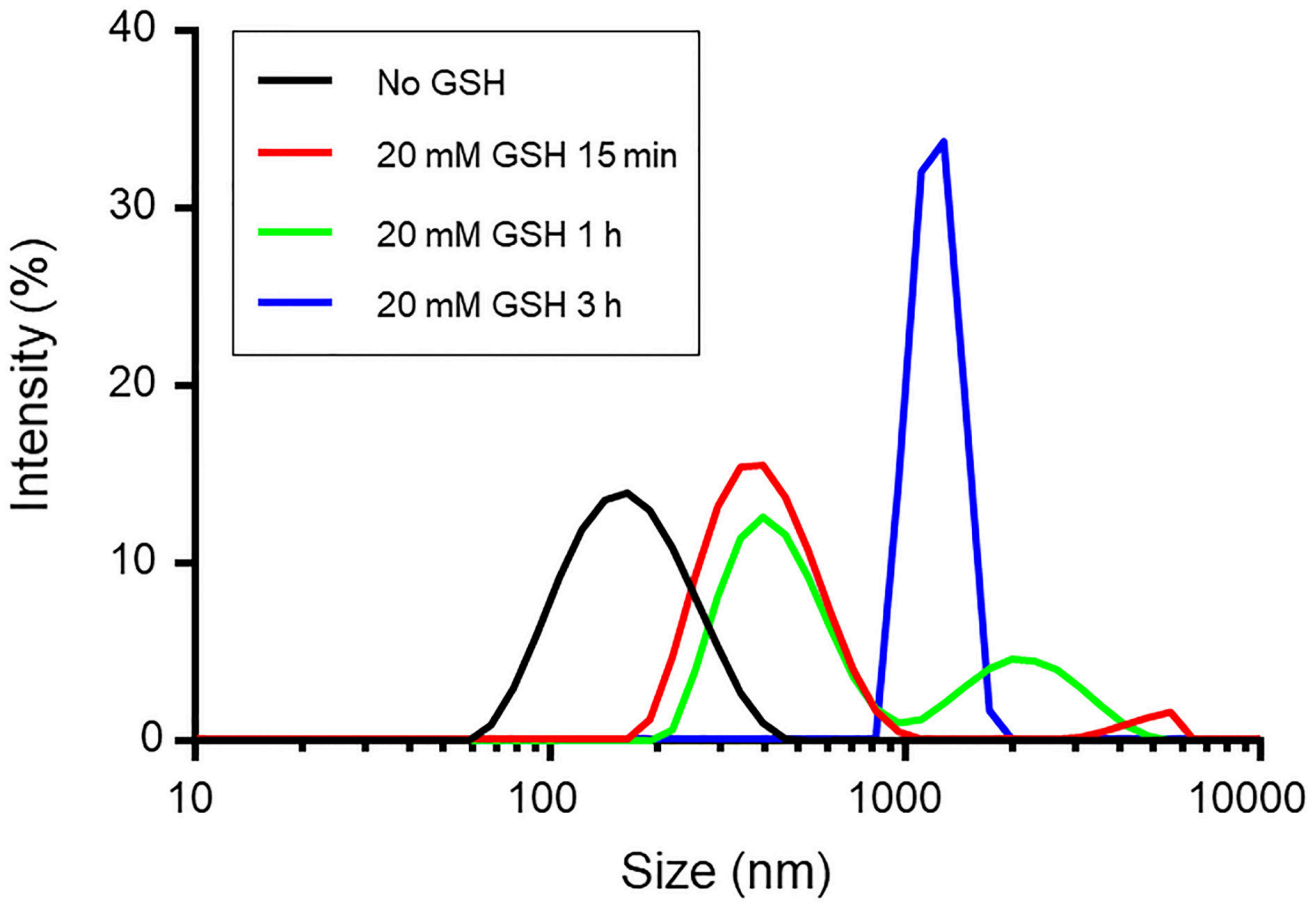
The size change of redox-responsive magnetic nanocarriers in response to 20 mM GSH in PBS determined by DLS measurement.

Disulfide linkages are known to be readily reduced into free thiols in the presence of reducing agents. To demonstrate the responsiveness, the size change of redox-responsive magnetic nanocarriers in response to 20 mM GSH in PBS was measured by DLS. Figure 2 shows that the average size of the nanocarriers gradually increased within the first 15 min after the addition of GSH. The size increased from 131 to 340 nm in 15 min, indicating the detachment of hydrophilic PEG shells from the nanocarriers and the enhanced hydrophobic interaction of the inner core. After 1 h, two populations at 547 nm and 1,038 nm were observed, however, after 3 h, the complete destruction of the nanocarriers was observed, and no nanoparticles were detected in the solution.

### *In vitro* Redox-Responsive DOX Release

**Figure 3:**
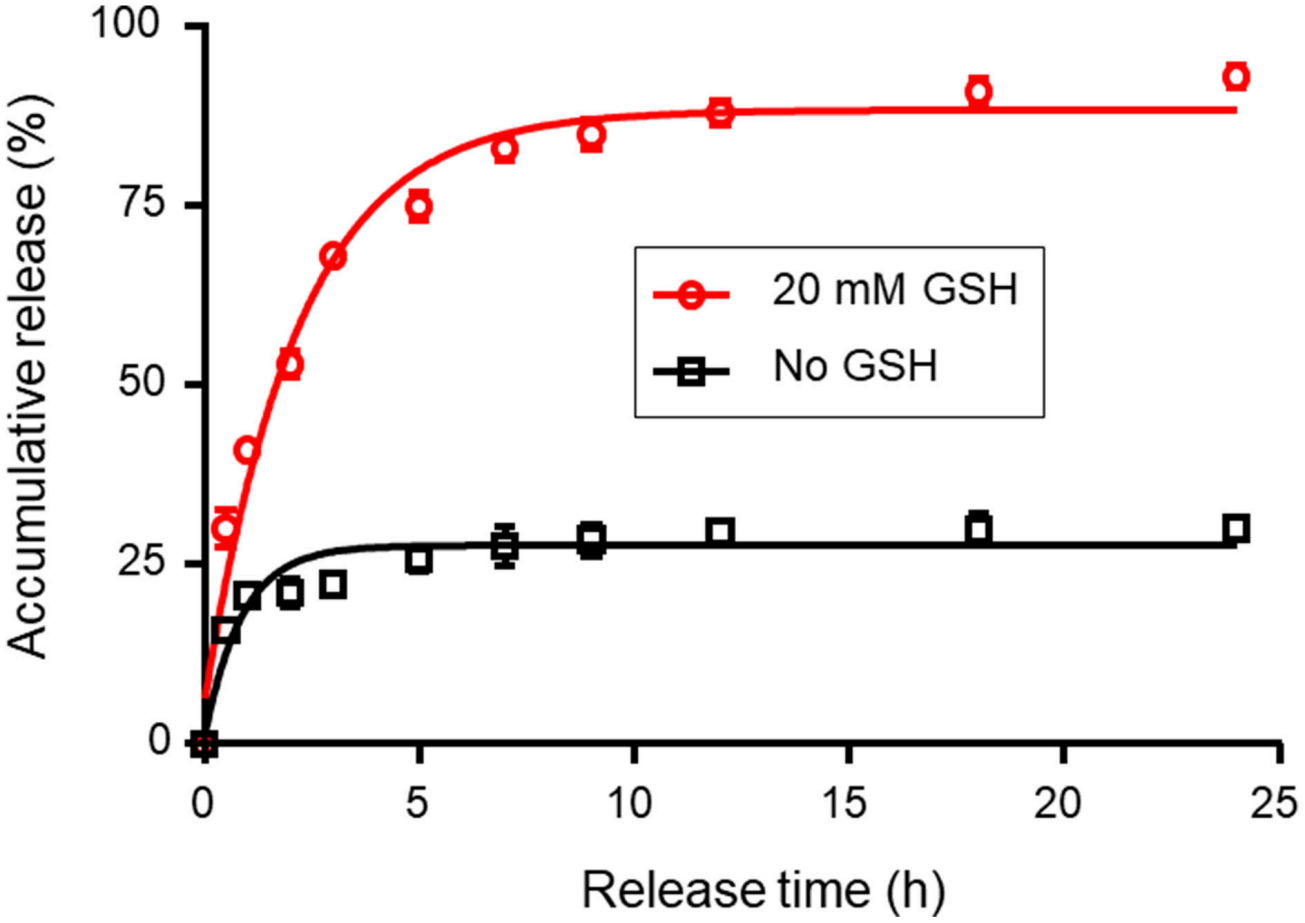
Redox-triggered release of DOX from redox-responsive magnetic nanocarriers in PBS with or without 20 mM GSH. The standard deviation for each data point was averaged over three samples (*n* = 3).

The drug release behavior of the DOX-loaded nanocarriers was investigated in PBS at 37°C in the presence or absence of GSH (20 mM). Figure 3 shows the accumulative drug release profiles as a function of time. Figure 3 demonstrates that the release of DOX from nanocarriers was markedly correlated with the presence or absence of GSH. The release of DOX from nanocarriers was accelerated by the addition of GSH to the media. In the presence of 20 mM GSH, nanocarriers rapidly released DOX, such that 93.8% of the DOX dose was released within 24 h. However, only 28.7% of DOX was released in the absence of GSH. This difference might be due to cleavage of disulfide bonds, thereby causing the destruction of the nanocarriers and the accelerated release of encapsulated DOX.

### Cell TEM Imaging

**Figure 4:**
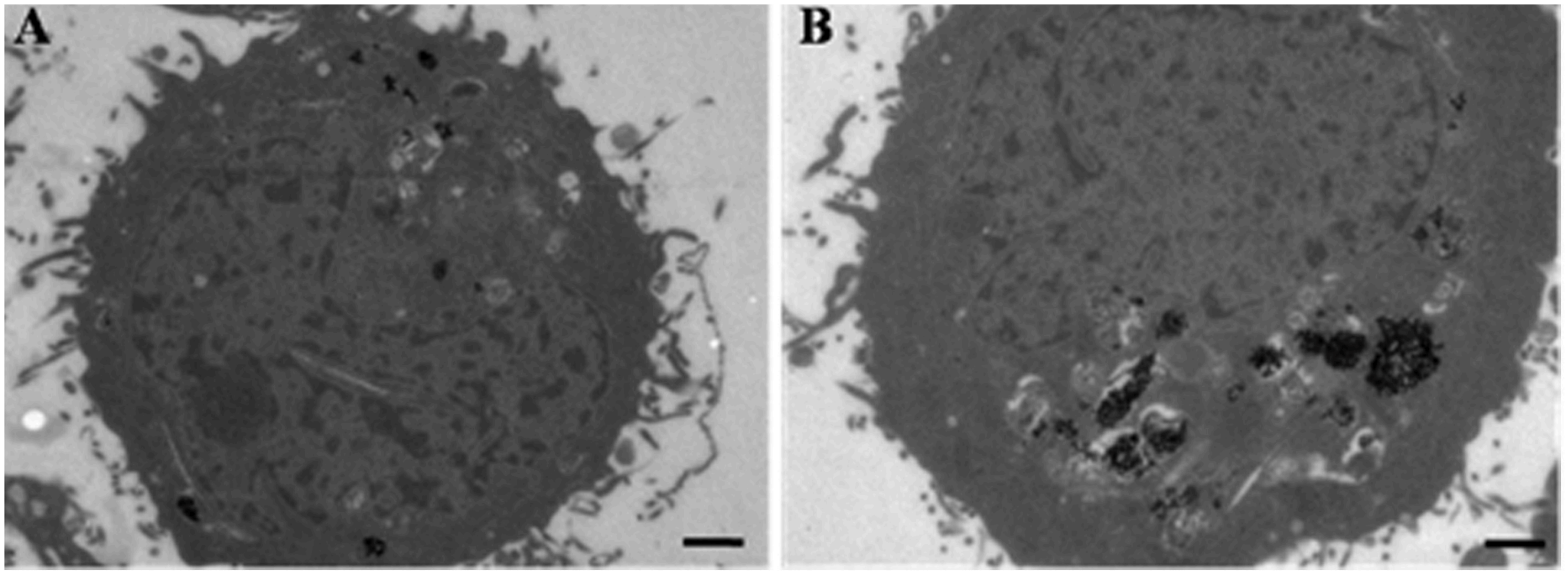
TEM images of HeLa cells incubated with DOX-loaded nanocarriers in the absence **(A)** or presence **(B)** of a magnetic field. Scale bar is 1 μm.

The rapid accumulation of DOX-loaded nanocarriers was found to be magnetically inducible *in vitro* and was characterized using TEM. When HeLa cells were incubated for 2 h with DOX-loaded nanocarriers in either the presence or absence of a magnetic field, the accumulation of nanocarriers was found to be altered. TEM images demonstrating this observation are shown in Figure 4. The heavily electro-dense iron-containing nanoparticles are reproduced in the TEM images as a significantly darker region in contrast to the cellular environment, which facilitated identification of relative particle density between groups. The number of magnetic nanoparticles in cells significantly increased when a magnetic field was applied (Figure 4B), suggesting that the presence of a magnetic field enhanced the accumulation of nanocarriers in cells.

### Cell Viability Assay

**Figure 5:**
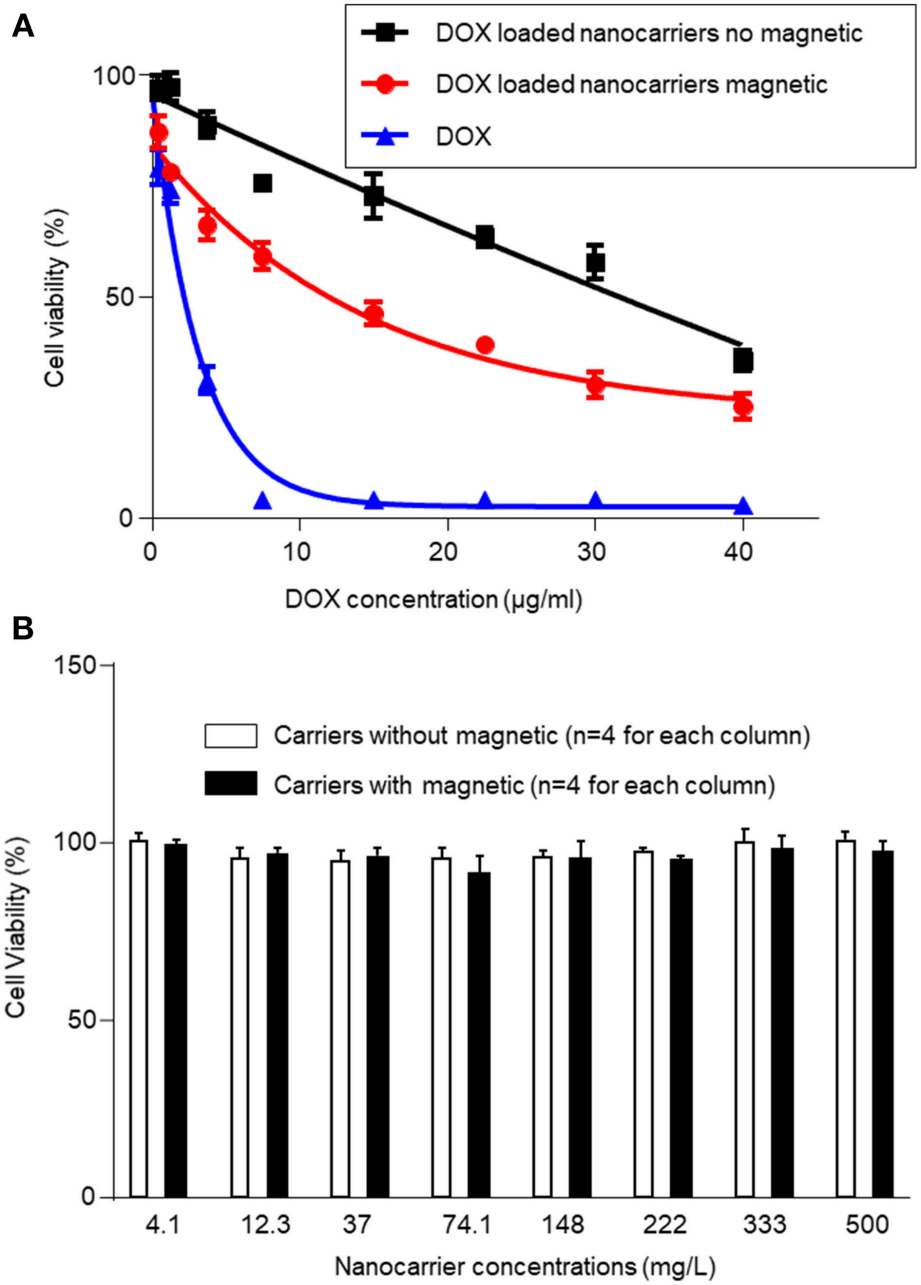
Cytotoxicity of DOX-loaded magnetic nanocarriers and free DOX in HeLa cells with or without magnetic field after incubation for 24 h **(A)**. **(B)** In control experiments, we examined cytotoxicity using magnetic nanocarriers alone with or without magnetic field after incubation for 24 h, and found no cytotoxicity. The standard deviation for each data point was averaged over four samples (*n* = 4) for **(A,B)**.

The *in vitro* cytotoxicity of DOX-loaded nanocarriers and free DOX was evaluated using the MTT assay. Figure 5 shows the resulting levels of observed cytotoxicity measured as a function of DOX concentration from 0.4 to 40 μg/mL. All test conditions exhibited a dose-dependent cytotoxic effect of the treatment on the population of viable and metabolically active HeLa cells. DOX-loaded nanocarriers exhibited lower cytotoxicity to HeLa cells with or without the magnetic field, as compared to free DOX at the same DOX dose (Figure 5A). As a control experiment, we performed a group of experiments using nanocarriers without DOX and examined cytotoxicity under magnetic and non-magnetic conditions, suggesting that the nanocarriers alone did not exhibit cytotoxicity (Figure 5B). Given that DOX is a small molecule, it can be quickly transported into cells and enter nuclei by passive diffusion. Furthermore, we found that the presence of a local magnetic field could significantly increase the cytotoxicity of DOX-loaded nanocarriers. This process may be due to the magnetic field increasing cellular uptake of nanocarriers, and once internalized, the redox-responsive nanocarriers are destroyed by high levels of GSH. The DOX is then rapidly released from the destroyed redox-responsive nanocarriers. Taken together these results indicate that the DOX-loaded nanocarriers can achieve both magnetic targeting and reduction-sensitive release simultaneously.

### *In vitro* Cellular Uptake of DOX-Loaded Nanocarriers

**Figure 6:**
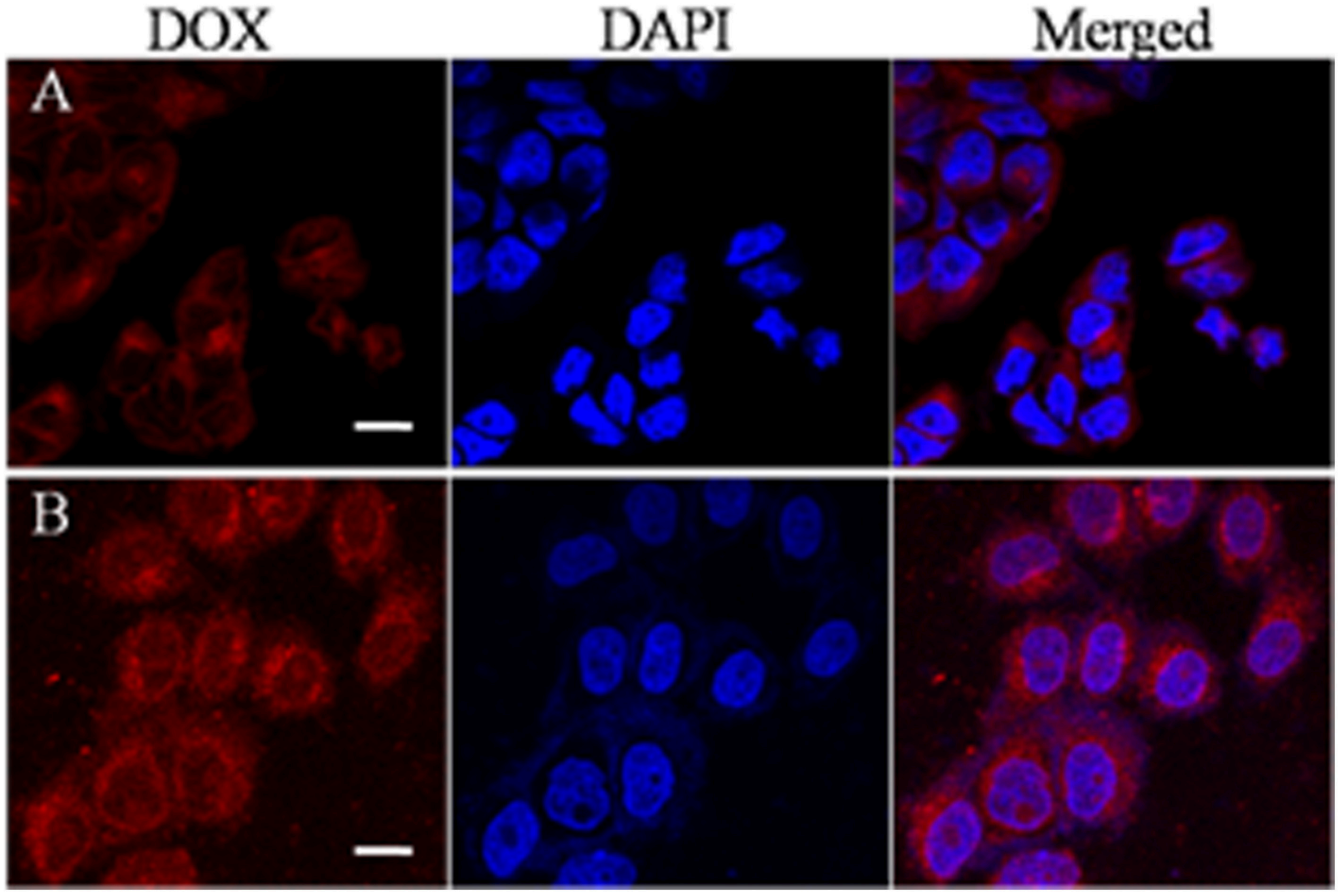
CLSM images of HeLa cells after treatment with DOX-loaded magnetic nanocarriers for 2 h in the absence **(A)** or presence **(B)** of a magnetic field. Scale bar is 15 μm.

The cellular uptake of the nanocarriers and the intracellular location of the encapsulated DOX was monitored by CLSM in HeLa cells. The nuclei of HeLa cells were stained with DAPI, which presented blue fluorescence to distinguish from the red fluorescence of the labeled DOX. Figure 6 shows CLSM images of HeLa cells incubated with DOX-loaded nanocarriers for 2 h with or without magnet field treatment. As shown in Figure 6, we found that cells incubated with DOX-loaded nanocarriers with applied magnetic field demonstrated stronger DOX fluorescence compared to no applied magnetic field. This phenomenon is primarily a result of the magnetic field increase in the cellular uptake of the DOX-loaded nanocarriers. Our results indicate that these nanocarriers are responsive to either magnetic or redox stimulated activation and are therefore suitable for application as anticancer drug carriers.

## Conclusion

In this article, we use the amphiphilic copolymer DSPE-SS-mPEG, which is connected by disulfide bonds. Afterward, the magnetic Fe_3_O_4_ nanoparticles and the hydrophobic drug are made by the self-assembly of the amphiphilic copolymer. DOX is encapsulated in the amphiphilic copolymer to form a magnetic nano drug controlled release system which is sensitive and responds to a reducing environment. This controlled release system can dissociate the disulfide bonds in the presence of dithiothreitol, thereby triggering the release system to disintegrate and expel the drug.

When the DOX-loaded nanocarrier is transported into the cell, intracellular GSH breaks the disulfide bonds, resulting in the disintegration of the transport system and the release of DOX. It is a well-designed enzyme-responsive magnetic-field controlled release system and provides a new foundation for building an efficient and safe nanoscale drug delivery system.

## Author Contributions

KX and YL equally contributed to this work, conducted the experiments, analyzed data, prepared original figures, and revised the manuscript. WY and GW participated in experimental design, analyzed the data, and revised the manuscript. HS analyzed the data and revised the manuscript. SZ designed the experiments and revised the manuscript. JW designed the experiments and analyzed the data, finalized the figures, and wrote the manuscript.

### Conflict of Interest Statement

The authors declare that the research was conducted in the absence of any commercial or financial relationships that could be construed as a potential conflict of interest.
